# Prevalence and factors associated with frailty among older adults with and without HIV in Kampala, Uganda

**DOI:** 10.1186/s12981-025-00824-7

**Published:** 2025-12-08

**Authors:** Phoebe Mbabazi, Grace Banturaki, Faizo Ssekindi, Suzan Naikoba, Peter W. Hunt, Meredith Greene, Matteo Cesari, Harriet Mayanja-Kizza, Barbara Castelnuovo

**Affiliations:** 1 Research Department, Infectious Disease Institute, Makerere University College of Health Sciences, Kampala, Uganda; 2 Division of Experimental Medicine, Department of Medicine, University of California, San Francisco, San Francisco, CA USA; 3 Division of General Internal Medicine & Geriatrics, Indiana University School of Medicine, Indianapolis, IN USA; 4 Indiana University Center for Aging Research, Regenstrief Institute, Indianapolis, IN USA; 5 Department of Clinical Sciences and Community Health, University of Milan, Milan, Italy; 6 Department of Internal Medicine, Makerere University College of Health Sciences, Kampala, Uganda

**Keywords:** HIV, Aging, Frailty, Sub-Saharan Africa

## Abstract

**Background:**

Sub-Saharan Africa has the highest prevalence of older adults with HIV worldwide, a subgroup with an increased risk of incident age-related conditions, such as frailty. We investigated the prevalence and factors linked to frailty among older people (aged ≥ 60 years) with HIV (PWH) and age- and sex-matched people without HIV (PWOH) in Kampala, Uganda.

**Methods:**

Frailty was assessed using the frailty phenotype, proposed by Fried and colleagues, based on five criteria: unintentional weight loss, exhaustion, low physical activity, slowness, and weakness. We estimated the prevalence of frailty and pre-frailty and fitted a modified Poisson regression model to identify significantly associated factors.

**Results:**

A total of 749 participants (371 PWH and 378 PWOH), 49.5% women, with a mean age of 67 (standard deviation 6.0) years. PWH had a median time of antiretroviral therapy (ART) use of 17 years (interquartile range 12–19), and 94.6% of PWH had viral suppression (viral load < 50 copies/ml). PWH had a similar prevalence of frailty (15.1% vs. 13.5%, P-value 0.53) and prefrailty (45.2% vs. 43.1%, P-value 0.55) compared to PWOH. Frailty was associated with older age, female sex, having no partner, being underweight, presenting food insecurity, and depressive symptoms.

**Conclusion:**

In our study, older PWH and PWOH had a similar prevalence of frailty and prefrailty. This unexpected result may be attributable to the benefits of ART and may reflect substantial improvements in the clinical management of PWH.

## Introduction

There is an exponential rise in the number of older adults with HIV in low and middle-income countries, especially in sub-Saharan Africa, due to improved access to antiretroviral therapy (ART) [1, 2]. However, even with suppressive ART, older people with HIV (PWH) experience higher rates of age-related comorbidities compared to those without HIV [3, 4]. The changing age demographic of PWH highlights a need to explore the burden of these age-related conditions and incorporate their management into the care of older PWH, particularly in sub-Saharan Africa, where geriatric medicine is still underdeveloped [5].

Frailty, a condition linked to adverse health outcomes such as an increased risk of mortality [6], results from an inability to cope with stressors following the exhaustion of physiological reserves [7]. Frailty is common among PWH and may have an earlier onset, especially in advanced HIV, partly because of the dysregulated immune system [8, 9]. A systematic review reported a prevalence of 5–28% among PWH in studies done in predominantly high-income countries [10], while a more recent review among PWH aged ≥ 50 found a prevalence of 10.9% [11]. The scarce reports from sub-Saharan Africa indicate variable prevalence of frailty among older PWH [12]. When compared to persons without HIV, PWH in sub-Saharan Africa have a higher prevalence of frailty in some reports [13–15], and similar or even lower prevalence of frailty in others [16, 17]. In rural Uganda, we assessed frailty using a modified self-reported frailty phenotype during the COVID-19 pandemic and found similar rates of frailty and lower rates of pre-frailty among older PWH on ART compared to those without HIV [18].

Factors leading to frailty among PWH in sub-Saharan Africa may differ from those described elsewhere because of biological, clinical, social, and economic differences. While factors like older age, female gender, and low CD4 count are illustrated by studies from both sub-Saharan Africa and high-income countries [10, 13, 16, 19], other factors like central obesity, lipodystrophy, the presence of non-communicable diseases, and food insecurity are derived mainly from studies in high-income countries [20–24]. Low body mass index or being underweight is a common risk factor for frailty among PWH in reports from sub-Saharan Africa [13, 15, 25], which may be mediated by food insecurity [26]. ART is documented to cause immune restoration, leading to a decline in frailty rates in PWH [27, 28]. However, PWH have persistent chronic inflammation despite HIV viral load suppression, which may contribute to higher rates of frailty when compared to PWOH [29, 30].

We hypothesized that the prevalence of frailty in PWH may be higher than that in PWOH. We determined the prevalence and factors associated with frailty among older adults with and without HIV in an urban outpatient clinic in Kampala, Uganda.

## Methods

### Study setting and design

This cross-sectional study was conducted between March 2023 and March 2024 at the Infectious Disease Institute (IDI) clinic in Kampala, Uganda, a center of excellence for HIV treatment and prevention, with over 8,000 patients on ART. PWH were enrolled from the diagnosis and treatment of non-communicable diseases (NCDs) and geriatric syndromes in the HIV aging population in sub-Saharan Africa (HASA) cohort at IDI. The HASA cohort was established in 2020 with 500 participants aged 60 years or older (the age cut-off for older persons in Uganda, as defined by the United Nations) [31]. Participants in the HASA cohort were seen annually and screened for NCDs such as renal impairment and geriatric syndromes such as cognitive impairment and falls [32].

As previously described [33], HIV uninfected adults were recruited from the community in Kampala, within a 2 km radius of the residence of the participants of the HASA cohort. We utilised existing resident data to estimate the number of older persons aged ≥ 60 years in an area. We used systematic sampling with a sampling interval determined by the estimated number of older adults in an area, while matching potential participants by age and sex to the HASA participants. Potential participants were asked to come to the study site, where HIV counselling and testing were done before enrollment.

We included adults aged ≥ 60 years with HIV on ART attending the HASA cohort at IDI during the study period and HIV-uninfected adults aged ≥ 60 years from the community in Kampala who provided written informed consent. We excluded participants who lived outside a 40 km radius of the IDI clinic. All HASA cohort participants who met the selection criteria were consecutively enrolled.

### Sample size estimation

The formula below for comparing two proportions was used to obtain a sample size of 378 participants in each group [34].

  

$$ {\text{n }} = {\text{ }}\frac{{\left( {{\mathrm{Z}}_{{\alpha /{\mathrm{2}}}} + {\mathrm{Z}}_{\beta } } \right)^{{\mathrm{2}}} *\left( {{\mathrm{p}}_{{\mathrm{1}}} \left( {{\mathrm{1}} - {\mathrm{p}}_{{\mathrm{1}}} } \right) + {\mathrm{p}}_{{\mathrm{2}}} \left( {{\mathrm{1}} - {\mathrm{p}}_{{\mathrm{2}}} } \right)} \right)}}{{\left( {{\mathrm{p}}_{{\mathrm{1}}} - {\mathrm{p}}_{{\mathrm{2}}} } \right)^{{\mathrm{2}}} }} $$

We used a 95% confidence interval, power of 80%, prevalence (P_1_) of frailty in PWH in the HASA cohort of 9% [32] and a prevalence (P_2_) of frailty in PWOH of 4%. We applied an approximate difference in frailty in PWH and PWOH of 5% which was estimated from a South African study that reported a difference in frailty of 6.1% in PWH and PWOH (19.4% vs. 13.3%) [13].

### Study procedures

*Frailty*, the main outcome of interest, was defined using the frailty phenotype by Fried and colleagues [35], which consists of five domains, with a modification for low physical activity [36].

1. Shrinking, defined as self-reported unintentional weight loss of ≥ 4.5 kg (10 pounds) or documented weight loss of ≥ 5% in the past year.
2. Self-reported exhaustion, assessed using two items from the Center for Epidemiological Depression (CES-D) scale [37].
3. Slowness (gait speed) of two consecutive measurements over a distance of 4.57 m (15 feet), and the result determined according to sex- and height-specific cut-offs.
4. Weakness or grip strength measured using a hand dynamometer, and the result determined according to sex and body mass index (BMI) cutoffs.
5. Low physical activity, defined as participants who answered “limited a lot” when asked if their health limits strenuous physical activities.

The presence of at least ≥ 3 criteria was defined as frailty, 1–2 criteria as pre-frailty, and the absence of all 5 as non-frail.

### Other variables

*Socio-demographics and medical history* **-** Participants were interviewed to collect socio-demographic information, including age, gender, education level, level of income (from all sources), marital status, tobacco use, and alcohol consumption, assessed using the Alcohol Use Disorders Identification Test- Concise (AUDIT-C) questionnaire [38].

We documented all current medications, including herbal medicines and comorbidities. Hypertension and diabetes mellitus were considered for those on medication or diagnosed according to World Health Organization (WHO) guidelines [39, 40]. Renal impairment was diagnosed if the estimated glomerular filtration rate (GFR) was < 60 mL/min/1.73 m^2^ using the Chronic Kidney Disease (CKD) Epidemiology Collaboration 2009 equation, without applying the race correction factor [41]. Other comorbidities were obtained by self-report and validated with medical records when available. ART history was retrieved from the IDI clinic database and laboratory tests, including blood sugar, serum creatinine, and CD4/CD8 count, performed at the IDI core laboratory.

*Depression* screening was performed using patient health questionnaires (PHQ-2 and PHQ-9), which have been validated in Uganda [42, 43]. Patients with mild, moderate, and severe depression were considered to have depressive symptoms.

*Cognitive impairment* was assessed using the Montreal Cognitive Assessment (MoCA) with the addition of one point for < 12 years of education and a lowered cut-off of 24 to account for cultural differences [44, 45].

*Functional impairment* was assessed using the Lawton Instrumental Activities of Daily Living (IADL) Scale, which explores 8 domains. Participants scoring < 8 were considered to have an impairment [46].

*Nutrition* was assessed using the Mini Nutritional Assessment and categorized as normal, at risk of malnutrition, and malnourished [47].

*Food security* was assessed using the validated 8-item Food Insecurity Experience Scale [48]. Participants with a score of 1–3 were considered to have mild food insecurity, 4–6 moderate, and 7–8 severe food insecurity.

### Statistical methods

We described participants’ characteristics using frequencies and percentages for categorical variables, means and standard deviations for normally distributed continuous variables, and medians and interquartile ranges for non-normally distributed continuous variables. We compared HIV infected and uninfected participants using the chi-square test for categorical variables, the Student’s t-test for normally distributed continuous variables, and the Kruskal-Wallis test for non-normally distributed continuous variables. We determined the crude prevalence of frailty, pre-frailty, and frailty sub-scales in participants with and without HIV, and by gender. We used the modified Poisson regression model (because the prevalence of frailty was > 10% and logistic regression tends to over-estimate the coefficients) to estimate the correlates of frailty while adjusting for HIV status, age, sex, having a current partner, number of comorbidities, BMI, food insecurity, cognitive impairment, and depression. Variables with a P-value < 0.25 in the univariable models were included in the multivariable model. We also included the presence of comorbidities in the multivariable model because of their importance. We checked for correlation between frailty sub-scales and covariates, such as shrinking and BMI, and between exhaustion and depression, and found no significant correlation. We also conducted subgroup analyses to explore potential interaction effects by HIV status and gender. Analyses were performed using Stata Version 18.5, and P-values < 0.05 were considered significant.

## Results

### Characteristics of the study participants

As shown in Table 1, a total of 749 participants, comprising 371 PWH and 378 PWOH, with a mean age of 67 years (SD 6.0), were included in the study. By design, participants with and without HIV were comparable in age and sex. PWH were less likely to have a partner (43.9% vs. 54.5%, P-value 0.004) and had a smaller household size (5.0 vs. 5.7, P-value 0.003). PWOH had a higher mean Audit-C score (0.9, SD 1.8 vs. 0.5, SD 1.0; P-value < 0.001) and were more likely to have high-risk alcohol (9.8% vs 2.7%, P-value < 0.001). Additionally, PWOH were more likely to be overweight (56.2% vs. 42.2%, P-value < 0.001). On the other hand, a higher proportion of PWH had one or more comorbidities (82.2% vs. 71.7%, P-value < 0.001) and had a higher median number of non-ART co-medications (2.0 IQR 1.0, 3.0 vs. 1.0 IQR 0.0, 2.0). PWOH were less likely to be on treatment for comorbidities such as hypertension (58.9% vs. 98.7%, P-value < 0.001). PWH had a median time on ART of 17 years (IQR 12,19), and 94.6% were virologically suppressed (Table 2).

**Table 1:** Characteristics of older people with and without HIV in Kampala

Characteristic	All *N* = 749	PWH *n* = 371	PWOH *n* = 378	*P*-value
Age (years, mean, SD)	67.0 (6.0)	67.3 (4.8)	66.7 (6.9)	0.13
Female (n, %)	371 (49.5)	180 (48.5)	191 (50.5)	0.58
Education (n, %)				
No formal education	38 (5.1)	13 (3.5)	25 (6.6)	0.26
Primary	310 (41.4)	154 (41.5)	156 (41.4)	
Secondary	278 (37.2)	140 (37.7)	138 (36.6)	
Technical/University	122 (16.3)	64 (17.3)	58 (15.4)	
Income < 1 $ /day (n, %)	210 (28.1)	98 (26.4)	112 (29.8)	0.41
Currently have a partner (n, %)	368 (49.3)	163 (43.9)	205 (54.5)	0.004
Household size (mean, SD)	5.4 (3.1)	5.0 (2.6)	5.7 (3.5)	0.003
Lives alone (n, %)	47 (6.3)	21 (5.7)	26 (6.9)	0.54
Alcohol, AUDIT- C score (mean, SD)	0.7 (1.5)	0.5 (1.0)	0.9 (1.8)	< 0.001
Positive score (n, %)	47 (6.3)	10 (2.7)	37 (9.8)	< 0.001
Present or Past tobacco use (n, %)	144 (19.3)	77 (20.8)	67 (17.8)	0.30
BMI (n, %)				
<18.5 kg/m^2^	46 (6.2)	25 (6.8)	21 (5.6)	< 0.001
18.5–24.9 kg/m^2^	328 (43.9)	189 (51.1)	139 (36.9)	
≥ 25 kg/m^2^	373 (49.9)	156 (42.2)	217 (57.6)	
Waist circumference (mean, SD)	86.4 (14.7)	82.4 (13.1)	90.3 (15.1)	< 0.001
Hip circumference (mean, SD)	95.4 (28.8)	92.0 (38.6)	98.8 (12.9)	0.001
Waist Hip Ratio (mean, SD)	0.9 (0.1)	0.9 (0.1)	0.9 (0.1)	0.79
Comorbidities (n, %)				
Diabetes Mellitus	112 (15.0)	56 (15.1)	56 (14.8)	0.92
Hypertension	454 (60.6)	230 (62.0)	224 (59.3)	0.44
Renal impairment	163 (21.8)	125 (33.7)	38 (10.1)	< 0.001
Cancer	6 (0.8)	5 (1.3)	1 (0.3)	0.12
Stroke	16 (2.1)	6 (1.6)	10 (2.6)	0.33
Other CVS conditions	12 (1.6)	9 (2.4)	3 (0.8)	0.09
Asthma	19 (2.5)	13 (3.5)	6 (1.6)	0.09
Epilepsy	3 (0.4)	2 (0.5)	1 (0.3)	0.55
Mental health illness	11 (1.5)	6 (1.6)	5 (1.3)	0.74
Chronic Hepatitis B	9 (1.2)	9 (2.4)	0 (0.0%)	0.002
Active Tuberculosis	3 (0.4)	3 (0.8)	0 (0.0)	0.12
Arthritis	139 (18.6)	61 (16.4)	78 (20.6)	0.14
Liver cirrhosis	2 (0.3)	2 (0.5)	0 (0.0)	0.15
Post-TB lung disease	3 (0.4)	3 (0.8)	0 (0.0)	0.08
Number of Comorbidities (n, %)				
0	173 (23.1)	66 (17.8)	107 (28.3)	< 0.001
≥1	576 (76.9)	305 (82.2)	271 (71.7)	
On treatment for comorbidity (n, %)				
Hypertension	359 (79.1)	227 (98.7)	132 (58.9)	< 0.001
Diabetes Mellitus	92 (82.1)	51 (91.1)	41(73.2)	0.01
IADL impairment (n, %)	53 (7.1)	24 (6.5)	29 (7.7)	0.52
Nutrition (n, %)				
Normal	612 (83.0)	312 (84.1)	309 (82.0)	
At risk of malnutrition	112 (15.0)	56 (15.1)	56 (14.9)	0.07
Malnourished	15 (2.0)	3 (0.8)	12 (3.2)	
Depressive symptoms (n, %)	34 (4.5)	23 (6.2)	11 (2.9)	0.03
Cognitive impairment (n, %)	491 (65.6)	230 (62.0)	261 (69.0)	0.04
Co-medications (non-ART) (median, IQR)	1.0 (0.0, 2.0)	2.0 (1.0, 3.0)	1.0 (0.0, 2.0)	< 0.001
Hospitalisation in the past year (n, %)	81 (10.8)	31 (8.4)	50 (13.2)	0.03
Food insecurity (n, %)				
Food secure	355 (47.5)	183 (49.5)	172 (45.6)	0.09
Mild	189 (25.3)	100 (27.0)	89 (23.6)	
Moderate	95 (12.7)	45 (12.2)	50 (13.3)	
Severe	108 (14.5)	42 (11.4)	66 (17.5)	

PWH, people with HIV; PWOH, people without HIV; N, number; SD, standard deviation; IQR, interquartile range; AUDIT-C, Alcohol Use Disorders Identification Test- Concise; BMI, body mass index; CVS, Cardiovascular; TB, Tuberculosis; ART, antiretroviral therapy; IADL, instrumental activities of daily living

**Table 2:** HIV-related characteristics of older people with HIV in Kampala

Characteristic	PWH (*n* = 371)
Years on ART (median, IQR)	17 (12,19)
Current CD4 (median, IQR**)**	628.0 (468.0, 860.0)
Pre-ART CD4 (median, IQR)	158.0 (68.0, 231.0)
HIV viral load < 50 copies/ mL (n, %)	350 (94.6%)
WHO stage III-IV (n, %)	286 (77.1)
ART category (n, %)	
NNRTI based	39 (10.6)
DTG based	292 (79.6)
PI based	34 (9.3)
IM Cabotegravir/ Rilpivirine	2 (0.5)

PWH, people with HIV; ART, antiretroviral therapy; IQR, interquartile range; WHO, World Health Organisation; NNRTI, Non-nucleoside reverse transcriptase inhibitors; DTG, Dolutegravir; PI, protease inhibitor; IM, Intramuscular injection

#### Prevalence of frailty, pre-frailty, and frailty sub-scales

As shown in Table 3, PWH had a similar prevalence of frailty (15.1% vs. 13.5%, P-value 0.53) and pre-frailty (45.2% vs. 43.1%, P-value 0.55) as PWOH. Similarly, there were no statistically significant differences in the frailty sub-scales among PWH and PWOH, although PWH had a higher prevalence of shrinking, exhaustion, low physical activity, and low grip strength.

Females had a higher prevalence of frailty compared to males (20.4% vs. 8.2%, P-value < 0.001). Similarly, in the frailty sub-scales, females had a higher prevalence of shrinking, exhaustion, low physical activity, and slowness compared to males.

**Table 3:** Prevalence of frailty, pre-frailty, and frailty domains among older people with and without HIV in Kampala by HIV status and gender

	Total (*N* = 749)	PWH (*n* = 371)	PWOH (*n* = 378)	*P*-Value	Males (*n* = 378)	Females (*n* = 371)	*P*-value
Frail (%, 95% CI)	14.3 (11.9–17.0)	15.1(11.6–19.1)	13.5 (10.2–17.4)	0.53	8.2 (5.6–11.4)	20.4 (16.5–25.0)	< 0.001
Pre-frail	44.2 (40.6–47.8)	45.2 (40.1–50.5)	43.1(38.1–48.3)	0.55	42.9 (37.8–48.0)	45.6 (40.4–50.8)	0.45
Robust	41.5 (37.9–45.1)	39.6 (34.6–44.8)	43.4 (38.3–48.6)	0.29	48.9 (43.8–54.1)	34.0 (29.2–39.0)	< 0.001
Shrinking	13.6 (11.2–16.3)	14.2 (10.9–18.3)	13.0 (9.7–16.8)	0.59	10.8 (7.9–14.4)	16.4 (12.8–20.6)	0.03
Exhaustion	20.0 (17.2–23.1)	22.3 (18.2–27.0)	17.8 (14.0–22.0)	0.11	13.0 (9.7–16.7)	27.2 (22.8–32.1)	< 0.001
Low physical activity	29.1(25.9–32.5)	30.7 (26.1–35.7)	27.5 (23.1–32.3)	0.11	17.2 (13.5–21.4)	41.2 (36.2–46.4)	< 0.001
Weakness	32.0 (28.7–35.6)	32.6 (27.9–37.6)	31.5 (26.8–36.4)	0.74	34.4 (29.6–39.4)	29.6 (25.0-34.6)	0.16
Slow walking speed	10.6 (8.5–13.0)	10.6 (7.6–14.2)	10.6 (7.7–14.2)	0.99	5.1 (3.1–7.7)	16.2 (12.6–20.4)	< 0.001

PWH, people with HIV; PWOH, people without HIV; CI, confidence interval

Confidence intervals were calculated using the exact binomial method

### Factors associated with frailty

As shown in Table 4, being older (adjusted prevalence ratio APR 1.07, 95% CI 1.04–1.09, P-value < 0.001), female (APR 1.74, 95% CI 1.07–2.83, P-value 0.03), having no partner (APR, 1.95, 95% CI 1.16–3.28, P-value 0.01), being underweight (APR 2.17, 95% CI 1.27–3.72, P-value 0.005), food insecurity (APR 1.74, 95% CI 1.16–2.60, P-Value 0.007), and the presence of symptoms of depression (APR 2.59, 95% CI 1.63–4.13, P-Value < 0.001) were associated with frailty. HIV was not associated with frailty (APR 1.16, 95% CI: 0.82–1.65, P-value 0.39). We checked for an interaction between HIV status and risky alcohol use on frailty and found that although PWH with high-risk alcohol use were more likely to be frail, the interaction was not statistically significant. In the sub-group analysis, the association between the covariates and frailty did not differ meaningfully across gender or HIV status. In the gender-stratified model, the interaction between gender and the covariates did not yield statistically significant results, and similar results were obtained in the HIV-status stratified model.

**Table 4:** Prevalence ratios of risk factors for frailty in the study sample

	Unadjusted PR (95% CI)	*P*-value	Adjusted PR (95% CI)	*P*-value
HIV Status				
HIV Uninfected	Reference		Reference	
HIV Infected	1.12 (0.79–1.59)	0.53	1.16 (0.82–1.65)	0.39
Age	1.06 (1.04–1.09)	< 0.001	1.07 (1.04–1.09)	< 0.001
Gender				
Male	Reference		Reference	
Female	2.50 (1.69–3.70)	< 0.001	1.74 (1.07–2.83)	0.03
Currently have a partner				
Yes	Reference		Reference	
No	0.32 (0.21–0.48)	< 0.001	1.95 (1.16–3.28)	0.01
Number of comorbidities				
0	Reference		Reference	
≥ 1	1.10(0.71–1.68)	0.67	0.78 (0.49–1.12)	0.15
BMI				
<18.5	2.01 (1.14–3.55)	0.02	2.17 (1.27–3.72)	0.005
18.5–24.9	Reference		Reference	
≥ 25	1.04 (0.71–1.53)	0.83	1.31 (0.89–1.92)	0.17
Food insecurity				
Food secure	Reference			
Food insecure	2.22 (1.49–3.28)	< 0.001	1.74 (1.16–2.60)	0.007
Cognitive impairment				
Not Impaired	Reference		Reference	
Impaired	2.29 (1.44–3.63]	< 0.001	1.52 (0.93–2.48)	0.09
Depression				
No depression	Reference		Reference	
Depression	3.42 (2.24–5.24)	< 0.001	2.59(1.63–4.13)	< 0.001

PR, prevalence ratio; CI, confidence interval; BMI, body mass index

## Discussion

In this assessment of older people aged 60 years or older in Kampala, Uganda, participants with HIV had a similar prevalence of frailty and pre-frailty as those without HIV. Older age, being female, having no partner, being underweight, food insecurity, and the presence of depressive symptoms were associated with frailty. This result is similar to our findings in rural Uganda, where participants with HIV had the same prevalence of frailty as those without HIV [18]. Similarly, a study in South Africa reported a similar prevalence of frailty among older adults with and without HIV [16], and a recent one in Zimbabwe reported a lower prevalence of frailty in PWH compared to those without HIV [17]. In one of the earliest descriptions of frailty among PWH in North America, which considered participants who were not on ART, HIV was associated with high rates and premature onset of frailty [9]. This was confirmed in subsequent reports, including a study from sub-Saharan Africa that included some participants who were not on ART [13]. Some reports from sub-Saharan Africa still indicate a higher prevalence of frailty among PWH on ART compared to those without HIV, but comparing them with our findings is difficult because of differences in frailty screening tools: the Edmonton Frail Scale [14] and the brief frailty instrument (B-FIT-2) [15].

Our observation of similar rates of frailty in PWH and those without HIV may be partly due to the excellent viral suppression in the participants. This was also observed in a longitudinal study among intravenous drug users, where those with well-controlled HIV exhibited a similar prevalence of frailty as the HIV uninfected [10]. In the current era of long-term ART use and viral suppression, frailty rates among older PWH may be comparable to those without HIV. Additionally, improved access to health care for managing comorbidities may have contributed to the frailty rates observed in PWH. In sub-Saharan Africa, PWH have better access to health care and management of comorbidities as they receive HIV care compared to the HIV uninfected [49]. There is insufficient screening and management of non-communicable comorbidities; for example, in Uganda, only 7.7% of persons with hypertension are aware of their diagnosis [50]. In our study, 58.9% of the participants without HIV were undergoing treatment for hypertension compared to almost all (98.7%) of those with HIV. Survivorship bias may also potentially contribute to the lack of a difference seen between PWH and PWOH. Unlike PWOH, the older adults with HIV in this study had to survive the dramatic stress of untreated HIV 20 years ago, and it is possible that only the strongest and most resilient PWH survived.

In this study, older age was associated with frailty, as shown in many reports, including some from sub-Saharan Africa [13, 16, 17, 19]. Frailty increases with age as physiological reserves and compensatory abilities gradually decline. Similarly, being female is also a documented risk factor for frailty in several reports [16, 19] and is related to behavioural, psychosocial, and biological differences between males and females, such as a higher risk of comorbidities like arthritis, obesity, and depression in women, which contribute to frailty [51]. The finding that having no partner is a correlate of frailty has been reported elsewhere in sub-Saharan Africa [15, 19] and in a systematic review, which found that being unmarried (widowed, divorced, separated, and never married) is associated with physical frailty [52].

Being underweight or malnourished is a recognised risk factor for frailty, as shown in other reports from sub-Saharan Africa [13, 15, 25]. Food insecurity is associated with frailty in reports from high-income countries [24, 53], which has not been demonstrated in sub-Saharan Africa, as well as in our study. Of note, we found a high prevalence of food insecurity, with more than half (52.5%) of participants demonstrating some degree of food insecurity and 14.5% having severe food insecurity, similar to other reports from sub-Saharan Africa [54, 55]. However, there was no difference in the rates of food insecurity observed in persons with and without HIV, and no association between food insecurity and being underweight. Food insecurity may be an important contributor to frailty in sub-Saharan Africa, where it is highly prevalent, especially among older adults [54]. In rural Uganda, food insecurity was associated with poor HIV outcomes such as ART non-adherence and incomplete viral suppression, which could potentially increase the risk of frailty [56]. Depression is a recognised risk factor for frailty in several studies [22, 57, 58], and the relationship is said to be bidirectional. Persons with depressive symptoms are more likely to be frail, while frail persons are more likely to have depressive symptoms [59].

A strength of this study is that we assessed a large number of PWH and comparators without HIV who were matched by age and sex and selected from the same communities. Additionally, the study was done within a prospective cohort for which instruments were planned and designed to assess frailty using objective measures of grip strength and gait speed. However, the cross-sectional design of the study does not allow us to define cause-and-effect relationships. Despite the large amount of collected data, it is not possible to exclude residual confounding from factors such as the age of infection that could explain the findings differently.

## Conclusions

Older people with and without HIV in this study had a similar prevalence of frailty and prefrailty. This unexpected result may be attributable to the benefits of antiretroviral therapy and may reflect substantial improvements in the clinical management of PWH. Addressing nutrition, food security, mental health, and improving the management of comorbidities, particularly in PWOH, may contribute to healthy aging in this population.

## Data Availability

The datasets used and/or analysed in the current study are available from the corresponding author upon reasonable request.

## Abbreviations

ART: Antiretroviral therapy
BMI: Body mass index
CES-D: Centre for Epidemiological Studies Depression Scale
CKD: Chronic kidney disease
GFR: Glomerular filtration rate
HASA: HIV Aging population in sub-Saharan Africa cohort
IADL: Instrumental Activities of Daily Living
IDI: Infectious Disease Institute
IQR: Interquartile range
MoCA: Montreal Cognitive Assessment
NCDs: Non-communicable diseases
PHQ: Patient health questionnaire
PWH: People with HIV
PWOH: People without HIV
SD: Standard deviation
SSA: Sub-Saharan Africa
TB: Tuberculosis
WHO: World Health Organization
